# Transcriptome-based epigenetic screening identifies DNA hypermethylation signatures as prognostic biomarkers in oral squamous cell carcinoma

**DOI:** 10.1186/s43556-026-00584-4

**Published:** 2026-09-17

**Authors:** Yu Kyeong Han, Jihye Park, Hye Su Han, Keunsoo Kang, Hae Ryoun Park, Joo Mi Yi

**Affiliations:** 1 Department of Microbiology and Immunology, College of Medicine, Inje University, Busan, 47392 South Korea; 2 Cardiovascular and Metabolic Diseases Medical Research Center, College of Medicine, Inje University, Busan, 47392 South Korea; 3 Department of Microbiology, Dankook University, Cheonan, 31116 South Korea; 4 Department of Oral Pathology, School of Dentistry, Pusan National University, Yangsan, Gyeongsangnam do 50612 South Korea

**Keywords:** Epigenetic regulation, DNA methylation biomarker, Promoter hypermethylation, Oral squamous cell carcinoma (OSCC), Prognosis

## Abstract

Promoter DNA hypermethylation is a key epigenetic mechanism of gene silencing in cancer, yet the DNA hypermethylome of oral squamous cell carcinoma (OSCC) and its prognostic relevance remain poorly characterized. Here, we systematically identified and validated novel hypermethylated genes with prognostic significance in OSCC using a genome-wide discovery and multi-platform validation strategy. Candidate genes were first identified by pharmacologic demethylation combined with RNA sequencing across OSCC cell lines, then validated by quantitative RT-PCR, methylation-specific PCR, and bisulfite sequencing in OSCC cell lines, normal oral mucosa, and primary OSCC tumors, with independent confirmation in the TCGA-HNSC dataset. Immunohistochemistry confirmed protein-level silencing, and Kaplan-Meier survival analysis assessed prognostic significance across both cohorts. This pipeline identified five candidate genes, *GPX3, ANG, CTGF, GPRC5B*, and *BAMBI*, exhibiting cancer-specific promoter hypermethylation associated with transcriptional and protein silencing in OSCC. Validation in oral cavity tumor samples extracted from the TCGA-HNSC dataset confirmed tumor-specific hypermethylation and revealed significant inverse correlations between methylation and expression for *GPX3, GPRC5B*, and *CTGF*. Notably, CTGF hypermethylation was independently associated with poor overall survival in both cohorts (institutional cohort, p=0.03; oral tumor subset from TCGA-HNSC, p=0.01), and a combined *ANG+CTGF* methylation signature showed superior and reproducible prognostic performance across both platforms. Pathway analysis linked these genes to epithelial-mesenchymal transition and interferon response signaling. This study establishes the first validated DNA methylation biomarker panel for OSCC prognosis, identifying *CTGF* hypermethylation as a robust prognostic driver with translational potential for clinical risk stratification.

**Supplementary Information:**

The online version contains supplementary material available at https://doi.org/10.1186/s43556-026-00584-4.

## Introduction

Head and neck squamous cell carcinoma (HNSC) is one of the most prevalent and lethal malignancies worldwide, with OSCC representing its most common subtype, accounting for over 90% of all oral cancers [1, 2]. OSCC is a highly aggressive disease characterized by frequent locoregional metastasis, high recurrence rates, and marked resistance to conventional chemotherapy and radiotherapy [3]. Globally, OSCC affects more than 350,000 individuals and claims approximately 170,000 lives annually [4, 5]. Despite significant advances in surgical techniques, radiation protocols, and targeted therapies, the 5-year survival rate for OSCC patients has remained at approximately 50–60% over the past several decades, underscoring the urgent need for improved diagnostic and prognostic tools [6, 7]. The lack of reliable molecular biomarkers capable of identifying high-risk patients at early stages remains one of the most critical unmet clinical needs in OSCC patient management.

Epigenetic alterations, particularly aberrant DNA methylation, have emerged as a hallmark of cancer cells and are now recognized as key drivers of tumor initiation and progression [8]. Unlike genetic mutations, epigenetic modifications are reversible, making them particularly attractive as both diagnostic biomarkers and therapeutic targets. In cancer cells, abnormal hypermethylation of CpG islands located within the promoter and enhancer regions of tumor suppressor genes (TSGs) leads to transcriptional silencing without altering the underlying DNA sequence, effectively mimicking the loss-of-function mutations seen in classical tumor suppressor inactivation [9]. This epigenetic silencing mechanism plays a well-established role in the inactivation of genes governing critical cellular processes including cell cycle regulation, apoptosis, DNA repair, and differentiation. Importantly, promoter hypermethylation events tend to occur early in carcinogenesis, often preceding morphological changes, making methylation-based biomarkers particularly promising for early detection and prognosis prediction [8, 9].

In OSCC specifically, aberrant promoter hypermethylation has been reported for a growing number of TSGs, including *p16, MGMT, RASSF1A, and DAPK*, among others [10–12]. However, the full landscape of hypermethylation-driven gene silencing in OSCC remains poorly characterized compared to other cancer types [13–16], and many epigenetically silenced genes with potential clinical relevance likely remain undiscovered. Genome-wide approaches to mapping the cancer methylome have proven powerful in revealing previously unknown hypermethylated loci, and integrating methylation data with transcriptomic profiling provides an especially sensitive and unbiased strategy for identifying genes whose silencing is driven by epigenetic mechanisms rather than mutational events [13].

Importantly, in DNMT1 and DNMT3B double knockout (DKO) colon cancer cells (HCT116), which exhibit near-complete loss of global 5-methylcytosine, previously characterized hypermethylated genes lacking basal expression in wild-type cells undergo promoter demethylation with concomitant gene re-expression [17, 18]. This genetic evidence underscores that pharmacological demethylation can serve as an effective proxy for uncovering epigenetically silenced genes. A particularly effective approach for this purpose involves restoring gene expression using DNA demethylating agents such as 5-aza-2′-deoxycytidine (decitabine; DAC, 5-aza-dC), a DNA methyltransferase inhibitor that causes passive demethylation of CpG islands during DNA replication [19]. Treatment of cancer cell lines with DAC reactivates genes silenced by promoter hypermethylation, enabling transcriptome-wide screening to systematically identify candidate methylation-regulated genes across the genome [13]. This strategy has been successfully applied across multiple cancer types to uncover novel epigenetically silenced tumor suppressor genes (TSGs) with diagnostic and prognostic relevance [14, 16]. However, its systematic application in OSCC has remained limited, leaving a substantial gap in our understanding of the OSCC-specific hypermethylation landscape and its clinical implications.

To address this gap, we performed genome-wide transcriptomic screening of a panel of five OSCC cell lines following DAC-induced demethylation treatment to identify genes reactivated by promoter CpG island hypermethylation. Through this approach, we identified a set of novel hypermethylated genes in OSCC that have not been previously reported in this cancer type. The methylation status and clinical significance of these candidate genes were further validated using TCGA-HNSC database and an independent cohort of patient samples. Our results demonstrate that the epigenetic silencing of these newly identified genes represents a biologically significant event in OSCC and that the resulting DNA hypermethylation signatures serve as clinically relevant biomarkers for patient prognosis. Collectively, this study expands the known landscape of epigenetic silencing in OSCC and provides a foundation for the development of novel methylation-based prognostic tools in oral cancer.

## Results

### Transcriptome-based screening identifies hypermethylated candidate genes in OSCC cells

To identify promoter hypermethylation-silenced genes, we employed a genome-wide pharmacologic strategy leveraging the well-established capacity of the DNA demethylating agent (5-aza-dC; DAC) to reactivate densely hypermethylated and transcriptionally silent genes. Critically, HDAC inhibitor trichostatin A (TSA) alone fails to induce re-expression of these same hypermethylated genes, and we exploited this divergent response to establish a stringent epigenetic selection criterion, selecting genes unresponsive to TSA yet robustly reactivated by DAC, to specifically enrich for bona fide DNA hypermethylation-silenced candidates [13]. This DAC-specific re-expression signature, observed as a characteristic spike of induced gene expression exclusively within the TSA-non-responsive zone, was validated against a genetic demethylation model and confirms that genes harboring dense CpG island promoter hypermethylation are selectively and reliably captured by DAC treatment alone.

**Fig. 1:**
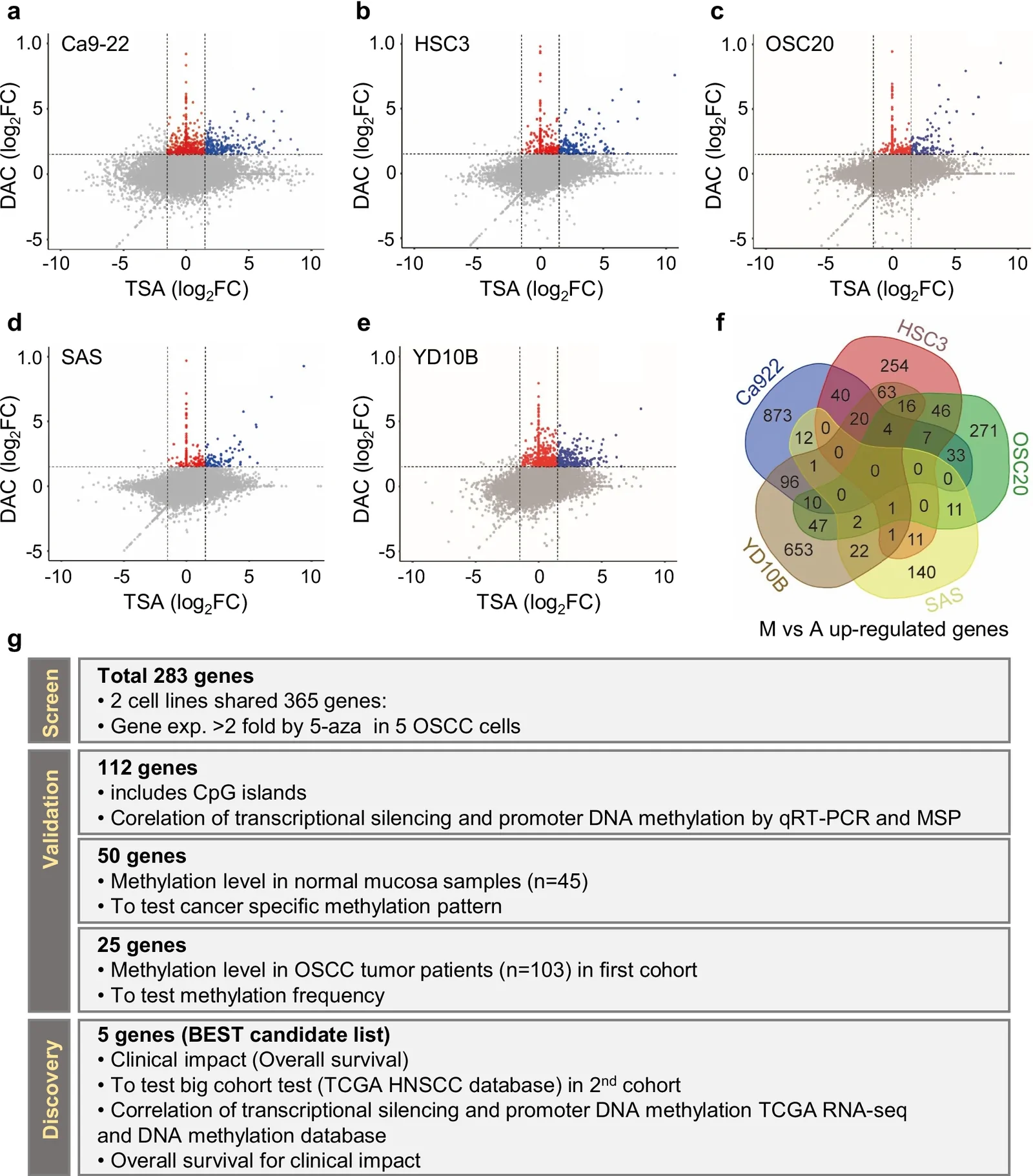
Genome-wide transcriptomic screening identifies promoter hypermethylation-silenced candidate genes in OSCC cell lines. **a**-**e** Gene-expression changes for the indicated cells treated with DAC (y-axis) or TSA (x-axis) are plotted by log-fold change, and individual genes are shown in grey. Genes are located in red was identified as a zone in which gene expression did not increase with TSA (,1.5 fold) and displayed no detectable expression in wild-type cells, but increased greater than 1.5-fold with DAC treatment. Blue region is genes are increasing expression level both by DAC and TSA treatment. **a**–**e** Scatter plots indicating genome-wide gene expression changes in response to TSA (x-axis, log_2_ fold change) and DAC (y-axis, log_2_ fold change) treatment in five OSCC cell lines: **a** CA9-22, **b** HSC3, **c** OSC20, **d** SAS, and **e** YD10B. Red dots represent genes exhibiting greater than 2-fold re-expression following DAC treatment with minimal response to TSA, representing bona fide promoter hypermethylation-silenced candidate genes. Blue dots represent genes re-expressed by TSA treatment. Gray dots represent genes that did not meet the re-expression criteria under either treatment condition. Dashed lines indicate the 2-fold change threshold. **f** Venn diagram illustrating the overlap of DAC-induced upregulated genes across the five OSCC cell lines, with numbers indicating genes unique to or shared between cell lines. **g** Schematic overview of the stepwise biomarker discovery pipeline, comprising three phases: screening (283 candidate genes exhibiting greater than 1.5~2-fold re-expression by DAC across five OSCC cell lines), validation (sequential filtering through CpG island annotation, qRT-PCR and MSP confirmation, normal mucosa methylation assessment, and tumor methylation frequency analysis), and discovery (identification of five best candidate genes and large-scale validation in the TCGA HNSC database including correlation of promoter DNA methylation with transcriptional silencing and overall survival analysis)

Applying this validated pharmacologic platform to the cancer-specific context of OSCC, we next implemented our genome-wide transcriptomic screen using RNA sequencing across five human OSCC cell lines (Ca9-22, OSC20, HSC3, YD10B, and SAS) to systematically discover genes silenced by aberrant promoter hypermethylation (Fig. 1a-e). By combining DAC-induced demethylation with TSA-based filtering, this platform selectively captures genes epigenetically repressed through CpG island promoter methylation, enabling high-confidence identification of disease-relevant candidates. Using this strategy, we identified a total of 2,634 unique candidate genes (Fig. 1f) across all five cell lines that fulfilled the criteria for promoter hypermethylation-associated silencing in OSCC.

To systematically identify and prioritize cancer-specific DNA methylation biomarkers in OSCC, we employed a stepwise pipeline encompassing genome-wide screening, multi-level validation, and discovery-phase characterization (Fig. 1g). In the screening phase, DAC-induced transcriptomic profiling across five OSCC cell lines identified 283 genes exhibiting greater than 1.5-fold re-expression (Table S1), with 365 genes shared across at least two cell lines, providing an initial pool of high-confidence hypermethylation candidates. In the validation phase, candidates were progressively refined through three sequential filters: (i) 112 genes harboring annotated CpG island promoters were selected and subjected to qRT-PCR and MSP to confirm transcriptional silencing and promoter DNA methylation (Table S2); (ii) 50 genes were subsequently assessed by MSP in normal oral mucosa samples (n=47) to evaluate baseline methylation and define cancer-specific patterns (Table S3a); and (iii) 25 genes were further analyzed for methylation frequency in a first cohort of OSCC tumor patients (n=103) (Table S3b). In the discovery phase, the top 5 candidate genes were further evaluated in the TCGA HNSC database as a second independent cohort. Comprehensive characterization of these genes, including correlation of promoter DNA methylation with transcriptional silencing by integrated RNA-seq and DNA methylation analysis, as well as overall survival assessment, is presented in the following result sections.

### Promoter DNA hypermethylation underlies transcriptional silencing of candidate genes in OSCC cell lines

**Fig. 2:**
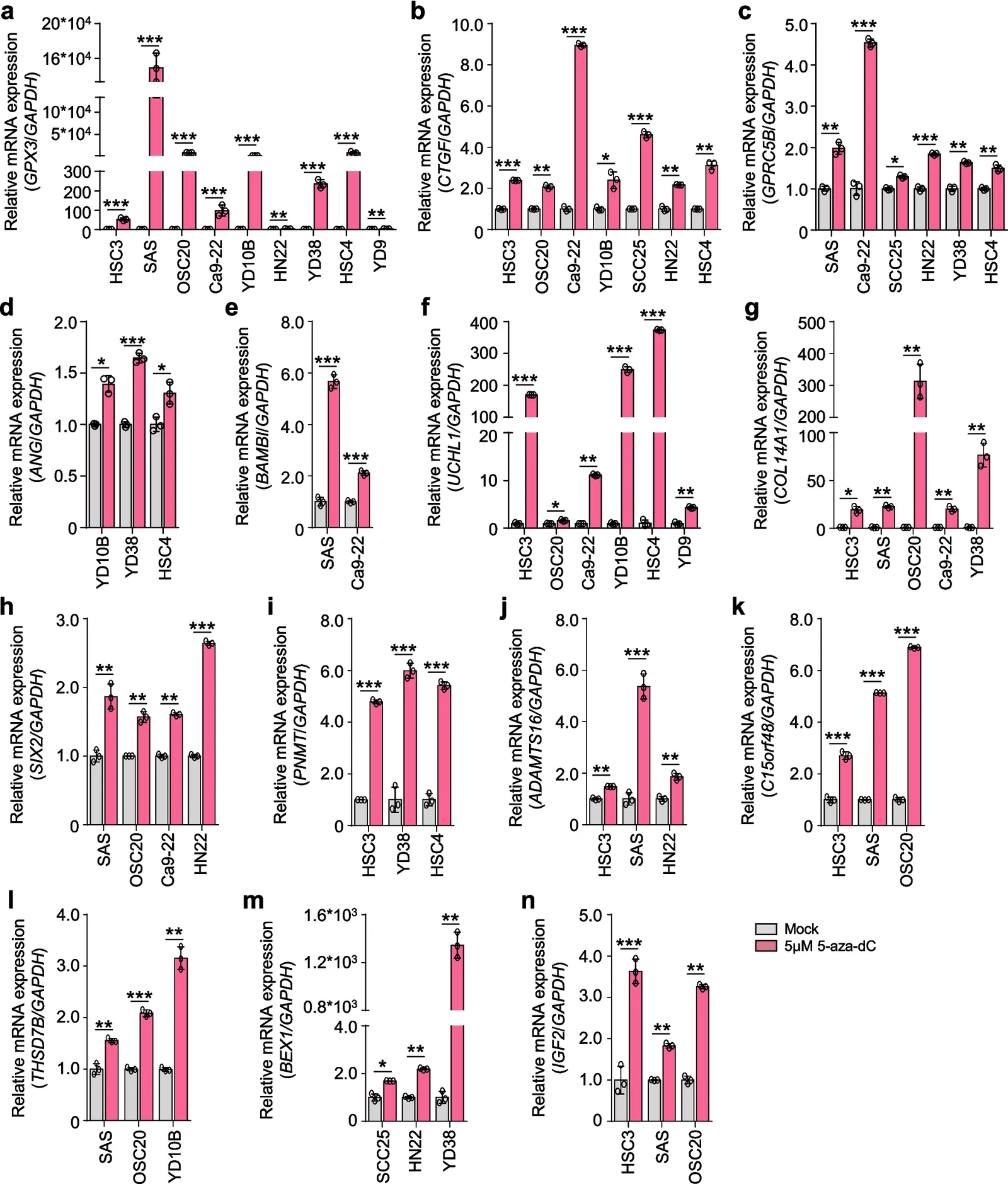
Epigenetic silencing and pharmacologic reactivation and promoter hypermethylation analysis of candidate genes in OSCC cell lines. Quantitative RT-PCR analysis of *GPX3, ANG, BAMBI, CTGF,* and *GPRC5B* expression was performed across 10 OSCC cell lines (Ca9-22, HSC4, OSC20, SAS, SCC25, HN22, YD10B, YD38, YD9, and HSC3) before and after treatment with 5 μM 5-aza-dC (DAC) for 72 hours. Expression levels were normalized to *GAPDH,* with the untreated condition for each gene set to 1. Statistical significance was assessed by a two-sided Student’s t-test, *p<0.05, **p<0.01, ***p<0.001, not significant. The data are presented as the mean ± SD for triplicate experiments. * indicates significant differences from control

**Fig. 3:**
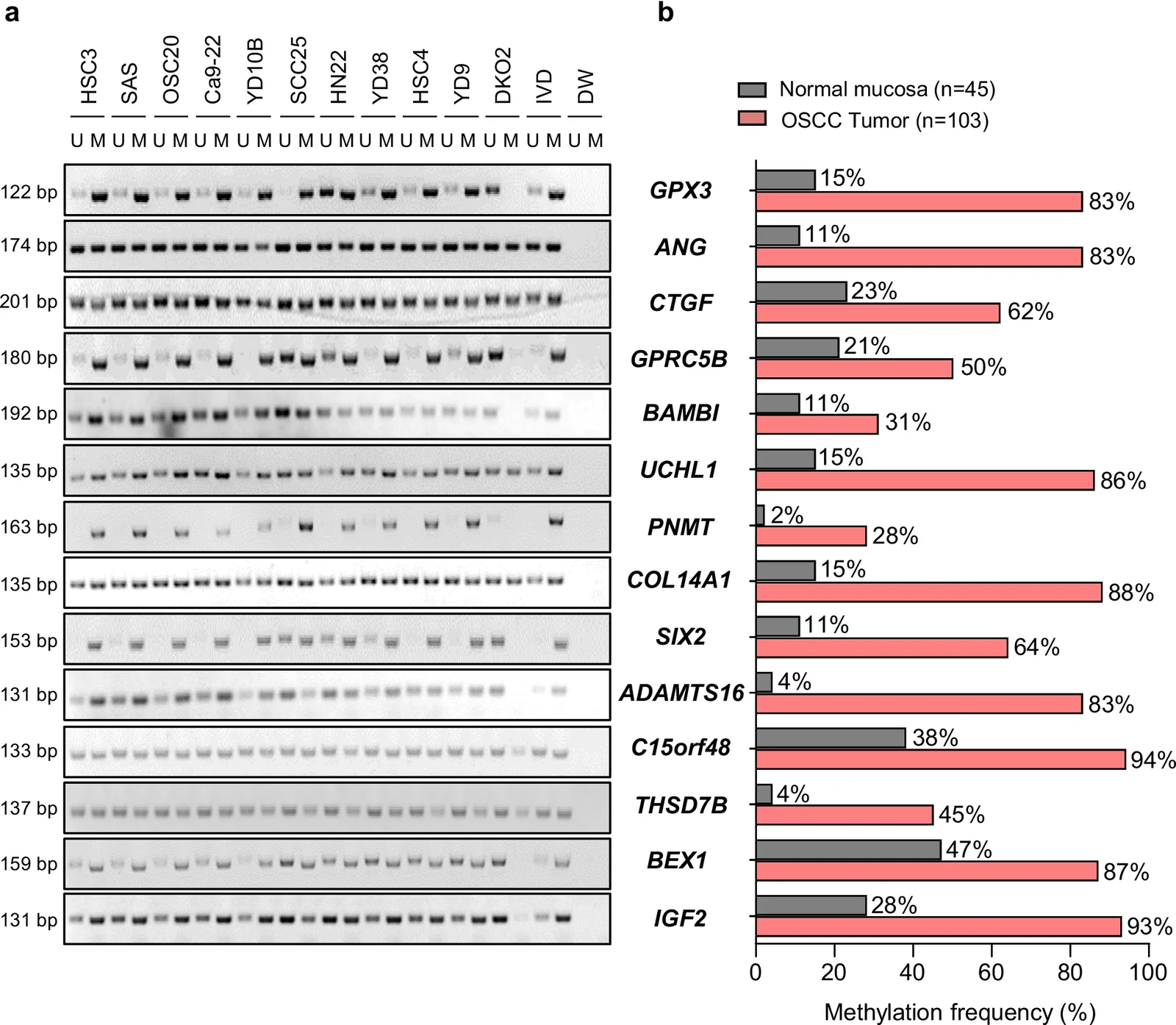
Promoter DNA methylation analysis of candidate genes in OSCC lines, normal oral mucosa, and primary OSCC tumor tissues. **a** MSP results of candidate genes in OSCC lines. Gel pictures describe the methylation analysis by methylation-specific PCR (MSP). DNA methyltransferase 1 and 3b knockout HCT116 cells (DKO) were included as positive controls. PCR products recognize unmethylated (U) and methylated (M). DKO cells were used for the unmethylated control. IVD = in vitro methylated control; ddH_2_O = water control containing no DNA. **b** Methylation frequency of the candidate genes in patients with OSCC (n = 103) and normal oral mucosa (n=47). Grey and pink bars indicate normal mucosa samples and OSCC tumor samples, respectively

To validate the candidate genes identified through our genome-wide RNA sequencing screening platform, we examined whether their transcriptional silencing is epigenetically regulated across 10 OSCC cell lines (Ca9-22, HSC4, OSC20, SAS, SCC25, HN22, YD10B, YD38, YD9, and HSC3) using RT-PCR. Cell lines were treated with 5-aza-dC to determine whether the loss of candidate gene expression could be restored through pharmacologic demethylation. As shown in Fig. 2a-e, *GPX3, CTGF,* and *GPRC5B* were transcriptionally silenced in the majority of OSCC cell lines and exhibited robust re-expression following 5-aza-dC treatment, predicting that their silencing is driven by promoter DNA hypermethylation. Additional candidate genes, including *ANG, UCHL, PNMT, COL14A1, SIX2, BAMBI, ADAMTS16, C15orf48, THSD7B, BEX1* and *IGF2,* met the re-expression criteria in at least two OSCC cell lines following 5-aza-dC treatment, even though it has more restricted pattern of silencing across the cell line panel (Fig. 2f-n). However, 5 candidate genes (*CDH2, VIM, WDR78, GRAMD1B,* and *DGKI*) failed to demonstrate consistent transcriptional silencing or statistically significant re-expression following 5-aza-dC treatment across the OSCC cell lines and were therefore excluded from further analysis. To directly test this prediction, we performed MSP in parallel with RT-PCR analyses to assess whether loss of expression correlates with promoter CpG island hypermethylation across the OSCC cell lines (Fig. 3a). This combined RT-PCR and MSP analysis established a direct link between promoter hypermethylation and transcriptional repression for each candidate gene.

### Clinical validation of promoter DNA hypermethylation in normal oral mucosa and OSCC patients

Having confirmed that promoter hypermethylation-associated silencing of the 14 candidate genes is a consistent feature across OSCC cell lines, we next sought to determine whether this epigenetic alteration extends to primary OSCC tumors and represents a cancer-specific event. We first examined the promoter methylation status of all candidate genes in normal oral mucosal tissues (n=45) to establish a cancer-specificity threshold (Fig. 3b). The majority of candidate genes exhibited methylation frequencies below 30% in normal tissues, supporting their classification as cancer-specifically methylated loci. However, three genes, *C15orf48, BEX1,* and *IGF2*, displayed high methylation frequencies in normal oral mucosa, indicating that their promoter methylation is not restricted to tumor cells and therefore does not represent a cancer-specific epigenetic alteration.

**Fig. 4:**
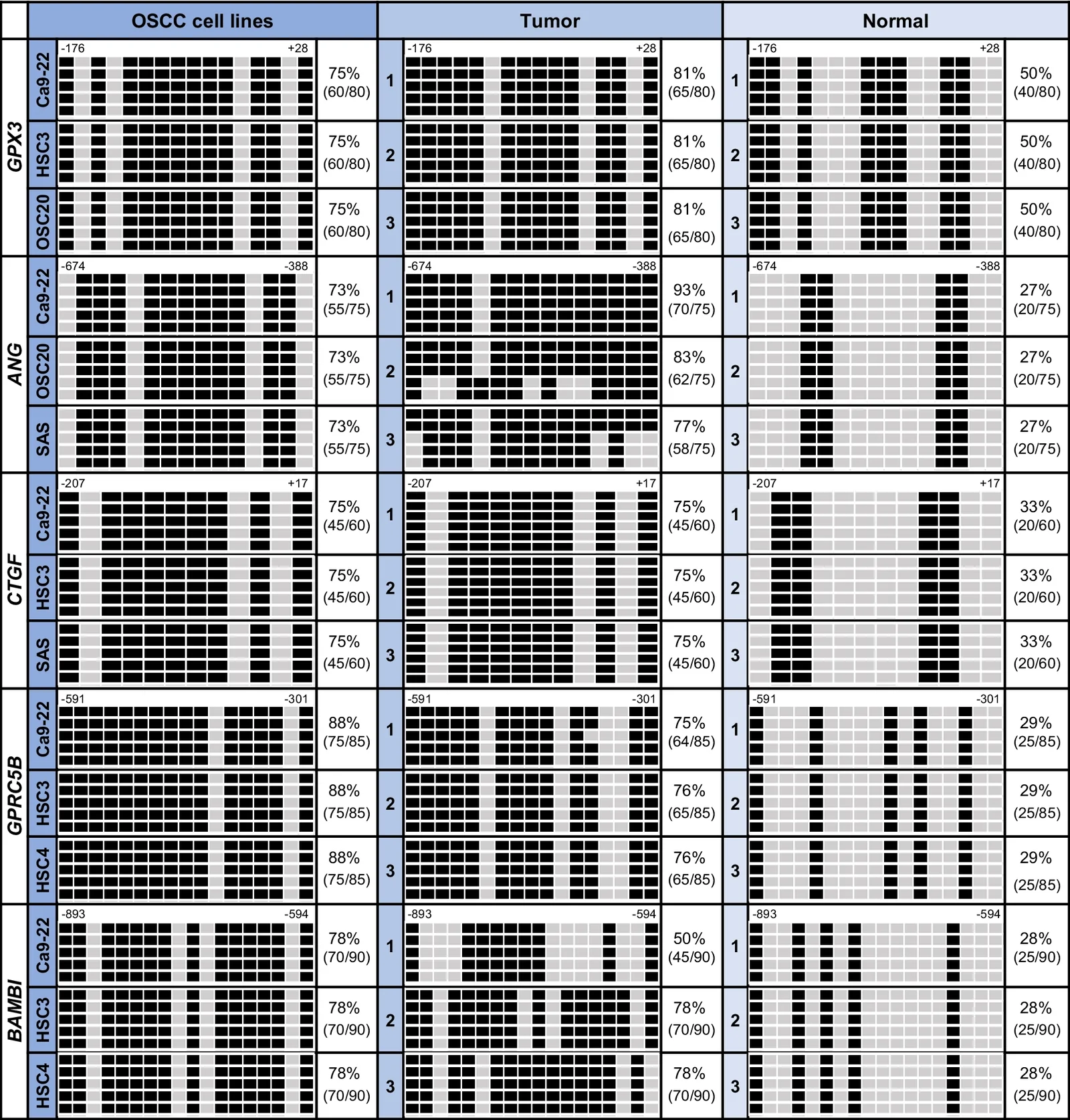
Bisulfite sequencing analysis of promoter CpG island methylation in OSCC cell lines, primary OSCC tumors, and normal oral mucosa. Representative bisulfite sequencing results are shown for the promoter CpG island regions of *GPX3, ANG, BAMBI, CTGF,* and *GPRC5B* in OSCC cell lines (n=3), primary OSCC tumor samples (n=3), and normal oral mucosal tissue samples (n=3). The analyzed CpG regions are defined relative to the transcription start site (TSS) of exon 1 as follows: *GPX3* (upstream region from −176 to +28, 16 CpG sites), *ANG* (−674 to −388, 15 CpG sites), *CTGF* (−207 to +17, 12 CpG sites), *GPRC5B* (−591 to −301, 17 CpG sites), and *BAMBI* (−893 to −594, 18 CpG sites). Each square represents an individual CpG dinucleotide, with filled squares indicating methylated cytosines and open squares indicating unmethylated cytosines; each row represents an individual sequenced clone. At least five clones were randomly selected and sequenced per candidate gene to determine the methylation pattern at each locus, with the total number of CpG sites analyzed calculated as the number of CpG sites per gene multiplied by five clones. The percentage of methylated CpG sites for each sample is indicated in the right panel

To assess methylation frequency in a clinically relevant context, we expanded our analysis to a large cohort of primary OSCC tumor specimens (n=103) [20]. Methylation-specific PCR revealed a broad range of promoter hypermethylation frequencies across the primary OSCC tumor samples (Fig. 3b), with methylation detected in 31–88% of tumors across the ten candidate genes, *COL14A1, UCHL1, GPX3, ADAMTS16, ANG, SIX2, CTGF, GPRC5B, THSD7B,* and *BAMBI*. However, five of these genes were subsequently excluded from further analysis on the basis that their promoter methylation could not be reliably confirmed at the sequence level in the OSCC cell lines, failing to meet the predefined validation criteria. The remaining five candidate genes, *GPX3, ANG, CTGF, GPRC5B*, and *BAMBI,* fulfilled all validation criteria and were advanced to bisulfite sequencing analysis for definitive confirmation of promoter CpG island hypermethylation at the sequence level (Fig. S1). Bisulfite sequencing analysis across OSCC cell lines and primary clinical specimens confirmed dense promoter CpG methylation for all five candidate genes. In OSCC cell lines, bisulfite sequencing revealed high levels of promoter CpG methylation in Ca9-22, HSC3, and OSC20 cells, with methylation levels of 73% in *ANG*, 75–76% in *GPX3, CTGF,* and *BAMBI*, and 88% in *GPRC5B* (Fig. 4), providing single-nucleotide resolution confirmation of the promoter hypermethylation pattern previously identified by MSP. To further validate these findings in a clinically relevant context, bisulfite sequencing was extended to representative primary OSCC tumor samples (n=3) and matched normal oral mucosal tissue samples (n=3). Primary OSCC tumors exhibited densely methylated promoter CpG sites across all five genes, with methylation levels of 81.3% in *GPX3*, 77–93% in *ANG*, 75% in *CTGF*, 75–76% in *GPRC5B*, and 53–76% in *BAMBI* (Fig. 4). In contrast, the corresponding normal oral mucosal tissues displayed markedly lower methylation levels at the same loci, *GPX3* at 50%, *ANG* at 27%, *CTGF* at 33%, *GPRC5B* at 29%, and *BAMBI* at 29%, demonstrating a striking differential methylation pattern between tumor and normal tissues. Taken together, these bisulfite sequencing data confirm cancer-specific promoter CpG island hypermethylation of all five candidate genes, fully corroborating our MSP results and establishing promoter hypermethylation as a key epigenetic mechanism underlying their transcriptional silencing in OSCC.

### Large-scale validation of candidate gene promoter hypermethylation in oral tumor samples extracted from TCGA-HNSC dataset

To confirm the relationship between promoter DNA methylation and transcriptional silencing of our candidate genes in a large independent cohort, we interrogated an oral cavity-specific subset curated from the TCGA-HNSC dataset (GDC TCGA-HNSC) (Table S4), excluding samples from non-oral anatomical subsites as described in the Methods, which provides matched DNA methylation (β values) and RNA-seq expression (FPKM) data across a large series of oral cavity tumor and normal tissue samples. Of the 11 candidate genes advanced to clinical validation, 6 genes were not retrievable in the TCGA HNSC database or failed to demonstrate a meaningful correlation between promoter DNA methylation and gene expression in this dataset and were therefore excluded from further TCGA-based analysis. The remaining five candidate genes, *GPX3, ANG, CTGF, GPRC5B,* and *BAMBI*, were consistently represented in the TCGA dataset with sufficient matched methylation and expression data, and were therefore selected for comprehensive large-scale validation.

**Fig. 5:**
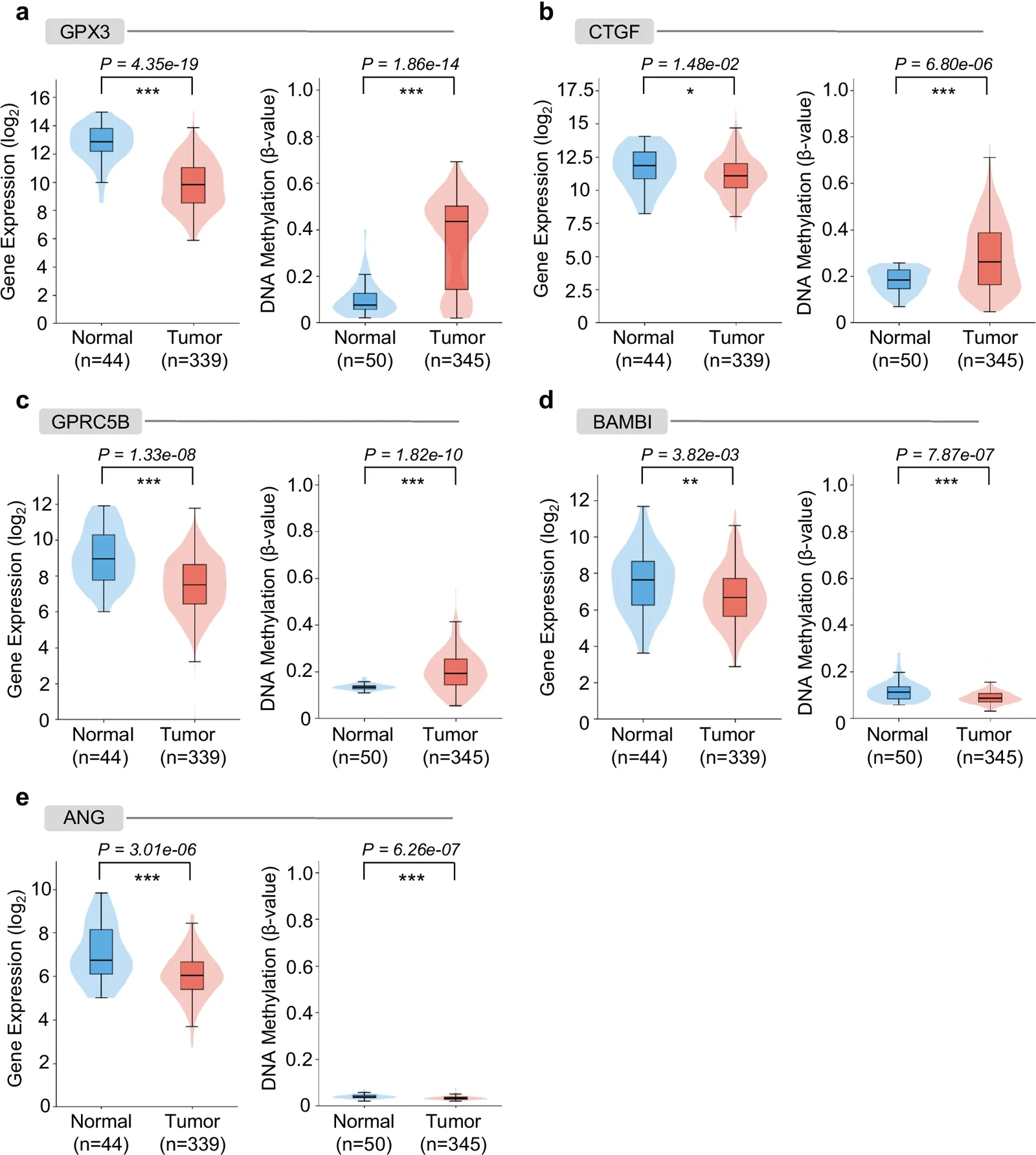
Correlation between promoter DNA methylation and gene expression levels of candidate genes in oral cancer patients extracted from the TCGA-HNSC dataset. Oral cancer-specific patient data were curated from the TCGA HNSC dataset for this analysis. (Left panel) Box plots comparing mRNA expression levels r **a** *GPX3,* **b** *ANG,* **c** *CTGF,* **d** *BAMBI*, and **e** *GPRC5B* between primary oral cancer tumor tissues (n=339) and normal oral mucosa (n=44). (Right panel) Box plots comparing promoter CpG site methylation levels (β values) of the same candidate genes between primary oral cancer tumor tissues (n=345) and normal oral mucosa (n=50). Statistical significance was determined by the Mann-Whitney U test for box plot comparisons

Box plot analysis revealed that all five candidate genes were significantly hypermethylated in HNSC tumor tissues compared to normal tissues, with a concomitant significant downregulation of mRNA expression, demonstrating a consistent inverse relationship between promoter DNA methylation and transcriptional activity across the cohort (Fig. 5). To further quantify this relationship, scatter plot analysis revealed a statistically significant inverse correlation between promoter DNA methylation and mRNA expression levels for three of the five candidate genes: *GPX3* (Fig. 5a; Spearman ρ = −0.621, p = 1.53 × 10⁻^3^⁷), *CTGF* (Fig. 5b; Spearman ρ = −0.155, p = 4.32 × 10⁻^3^), and *GPRC5B* (Fig. 5c; Spearman ρ = −0.384, p = 2.32 × 10⁻^13^) (Fig. S2a-c). Although *ANG* and *BAMBI* did not reach statistical significance in the correlation analysis (Fig. 5d, e; Fig. S2d,e), their consistent hypermethylation and transcriptional downregulation observed in the box plot analysis suggest that additional epigenetic or transcriptional regulatory mechanisms may contribute to their silencing in HNSC. Taken together, these TCGA-based findings are fully concordant with our experimental RT-PCR, MSP, and bisulfite sequencing data, collectively reinforcing promoter CpG island hypermethylation as a key epigenetic mechanism underlying transcriptional silencing of these candidate genes in OSCC and supporting their potential utility as epigenetic biomarkers in OSCC.

### Loss of protein expression confirms epigenetic silencing of candidate genes in primary OSCC tumors

**Fig. 6:**
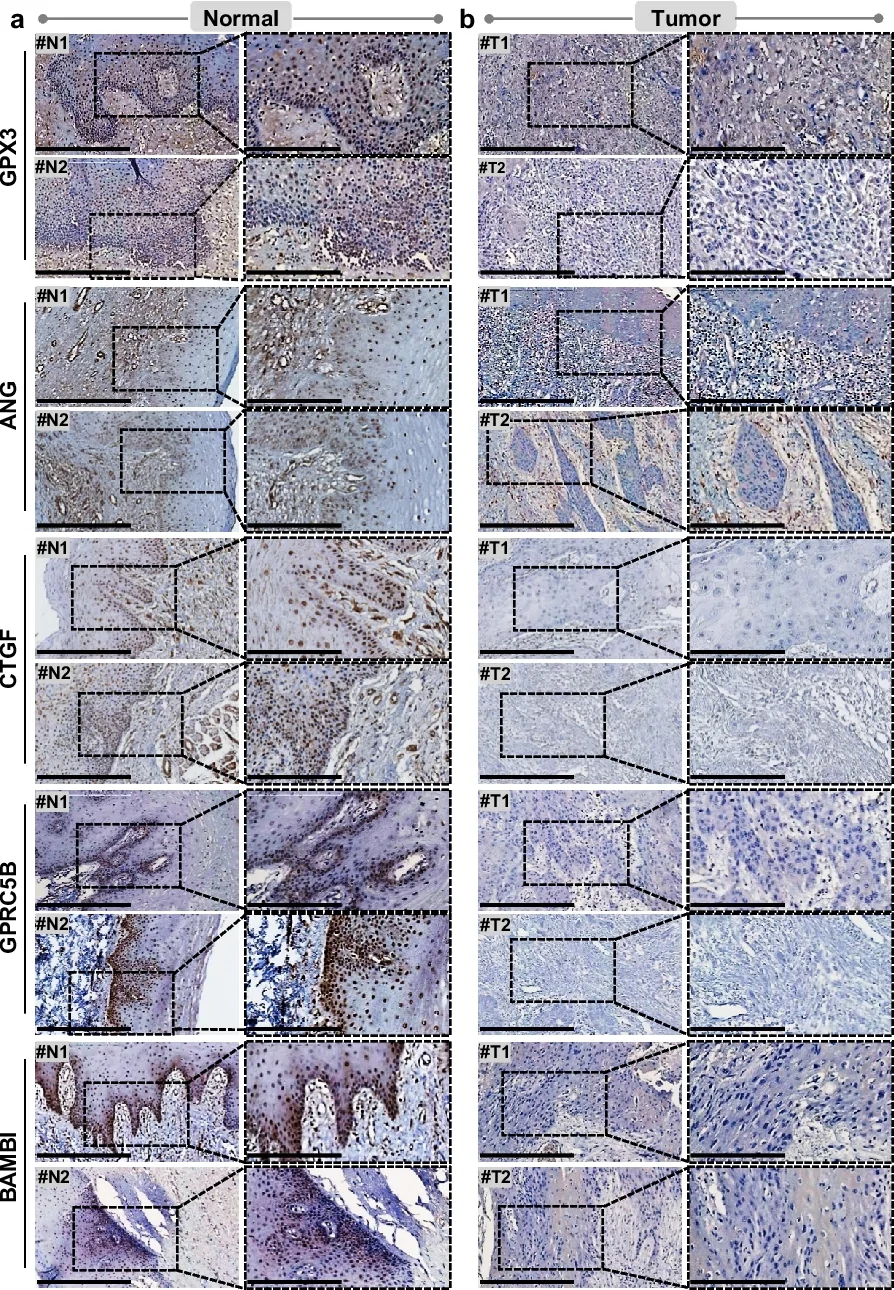
Protein expression levels of five genes in primary OSCC tumors and normal oral mucosa tissue samples. Representative immunohistochemical analysis results showing GPX3, ANG, CTGF, GPRC5B, and BAMBI expression in **a** normal oral mucosa and **b** primary OSCC tissues. Left images: × 20, scale bar, 200 µm; Right images: × 40, scale bar, 100 µm

To further validate the epigenetic silencing of the five candidate genes at the protein level, we examined the expression of GPX3, CTGF, BAMBI, GPRC5B, and ANG by immunohistochemical (IHC) staining in primary OSCC tumor tissues and matched normal oral mucosal tissues from our institutional cohort. Representative immunohistochemical staining images for the five candidate genes are shown in Fig. 6; comprehensive staining data for all cases are provided in Fig. S3. In normal oral mucosal tissues (n=2), strong positive staining was observed for all five proteins, confirming their robust expression under physiologically normal conditions. In contrast, primary OSCC tumor tissues (n=2) exhibited markedly reduced or absent protein expression for all five candidates compared to normal oral mucosa, consistent with the promoter hypermethylation-driven transcriptional silencing observed at the mRNA level (Fig. 6; Fig. S3). These findings collectively demonstrate that the epigenetic silencing of GPX3, CTGF, BAMBI, GPRC5B, and ANG is reflected at the protein level in primary OSCC tumors, further suggesting that the coordinated epigenetic inactivation of these genes may represent a hallmark molecular event in OSCC tumorigenesis with implications for early detection, prognostication, and epigenetic therapeutic intervention.

### Exploratory survival analysis suggests promoter DNA hypermethylation of candidate genes as a potential prognostic indicator in OSCC

To evaluate the clinical significance of promoter DNA hypermethylation of the five candidate genes, we interrogated the TCGA HNSC dataset, which included 528 primary tumor samples. After anatomic-site curation for OSCC-focused analysis, 344 oral cavity cases were retained, with 184 cases excluded based on non-oral cavity subsite designation (Table S4); one retained case lacked overall survival time, leaving 343 cases for survival analysis.

**Fig. 7:**
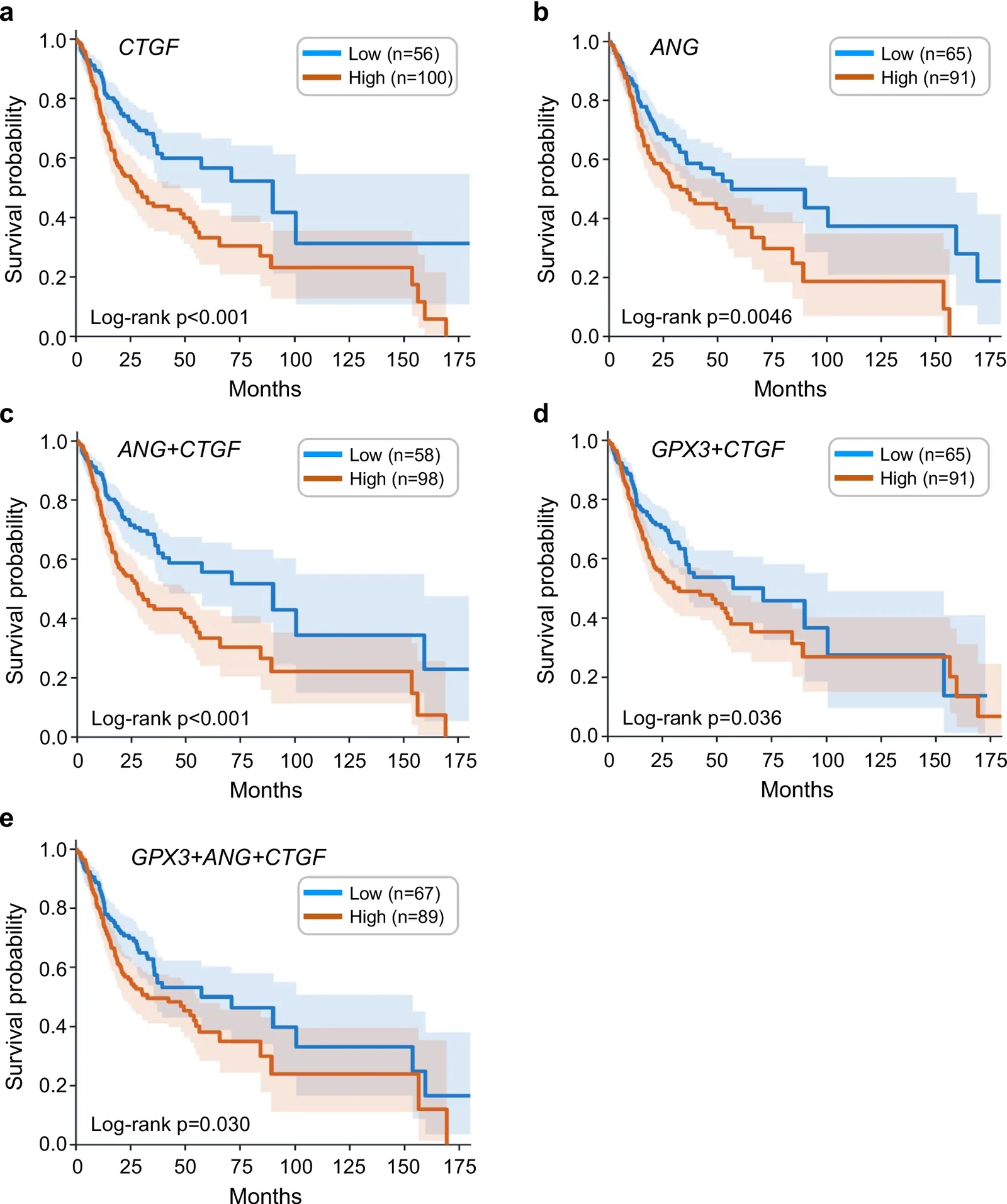
Exploratory Kaplan-Meier survival analysis of candidate gene promoter methylation in the curated TCGA-HNSC oral-cavity cohort. Of 344 retained oral-cavity cases, one lacked overall-survival time; 343 cases were included. The representative BEST CpG for each gene was selected before survival analysis as the promoter-window probe with the lowest tumor-versus-normal methylation Mann-Whitney P value, not according to overall-survival or log-rank P values. High and low groups were defined by the cohort median of the selected CpG beta value or, for gene combinations, by the median of the arithmetic mean beta value across the selected CpG features. **a** *CTGF* (cg24684168, low n = 171 (56 events) and high n = 172 (100 events), log-rank p = 7.95 x 10^−5.) **b** *ANG* (cg10850001, low n = 171 (65 events) and high n = 172 (91 events), log-rank p = 0.0046). **c** *ANG+CTGF* (mean beta score, low n = 171 (58 events) and high n = 172 (98 events), log-rank p = 9.81 x 10^−5). **d** *GPX3+CTGF* (mean beta score, low n = 171 (65 events) and high n = 172 (91 events), log-rank p = 0.036). **e** *GPX3+ANG+CTGF* (mean beta score, low n = 171 (67 events) and high n = 172 (89 events), log-rank p = 0.030). Shaded areas indicate 95% confidence intervals. P values were calculated using two-sided log-rank tests. These Kaplan-Meier analyses are exploratory; adjusted TCGA survival modeling is reported separately using Cox proportional hazards regression

In exploratory Kaplan-Meier analyses using fixed promoter CpG features selected by tumor-versus-normal methylation differences, not survival P-values, higher methylation was associated with poorer overall survival for CTGF (p < 0.001, Fig. 7a) and ANG (p=0.0046, Fig. 7b), while GPX3, BAMBI, and GPRC5B did not demonstrate meaningful prognostic associations (Fig. S4a–c). Multi-gene combination panels also showed exploratory associations, including *ANG+CTGF* (p<0.001, Fig. 7c), *GPX3+CTGF* (p=0.0364, Fig. 7d), and *GPX3+ANG+CTGF* (p=0.0299, Fig. 7e). Promoter-window analyses of average and best CpG-based promoter features, including the nominal Cox P-value distribution and within-screen FDR context for the fixed CpG features used, are provided in Fig S5.

To assess whether these associations represent independent prognostic effects, continuous methylation scores were evaluated using multivariable Cox proportional hazards regression adjusted for age, sex, and pathologic stage (primary model; n=311, events=143). In this model, *CTGF* methylation was not significantly associated with overall survival (HR=1.06 per 0.1 β-value increase, 95% CI 0.92–1.22, P=0.413). Although all individual genes and gene combination panels showed hazard ratios consistently above 1.0, suggesting a directional trend toward increased risk with higher methylation scores, none reached statistical significance under multivariable adjustment (Fig. S6a). Sensitivity models additionally incorporating HPV status, smoking history, nodal status, T category, and primary radiotherapy, applied to the same gene set evaluated in Fig 7, including individual genes and all combination panels, yielded the same overall interpretation, with none reaching statistical significance (Fig. S6b). Clinical covariate availability is summarized in Fig. S7. These results indicate that while the Kaplan-Meier associations are hypothesis-generating, the methylation scores evaluated do not demonstrate independent prognostic utility after adjustment for established clinical covariates in the TCGA cohort.

To independently assess CTGF promoter hypermethylation in a separate patient population, we performed Kaplan-Meier survival analysis in our institutional cohort (n=103), stratifying patients by MSP-based methylation status. CTGF promoter hypermethylation was significantly associated with poor overall survival in methylated compared to unmethylated tumors (p=0.030, Fig. S4d). The remaining four candidate genes did not demonstrate statistically significant survival associations in this cohort, likely reflecting limited statistical power given the smaller sample size. Additionally, several two-gene combination panels showed significant associations in the institutional cohort, including *ANG+CTGF* (p=0.047, Fig. S4e), *GPRC5B+CTGF* (p=0.044, Fig. S4f), and *ANG+GPRC5B* (p=0.048, Fig. S4g), while three-gene (*ANG+GPRC5B+CTGF*, p=0.054) and four-gene combinations (*ANG+GPRC5B+CTGF+BAMBI*, p=0.058) showed trends toward poor survival without reaching statistical significance (Fig. S4h–i).

Taken together, these findings suggest that *CTGF* promoter hypermethylation is a candidate prognostic marker in OSCC, with consistent directional associations observed across two independent cohorts and distinct methylation detection platforms. However, as the TCGA-based association did not remain significant under multivariable adjustment, *CTGF* methylation should not yet be considered an independently validated prognostic biomarker, and these findings should be regarded as hypothesis-generating, warranting prospective validation in larger, well-annotated cohorts using prespecified statistical approaches.

### Functional pathway enrichment analysis implicates epigenetic silencing in cancer-associated signaling pathways in OSCC

**Fig. 8:**
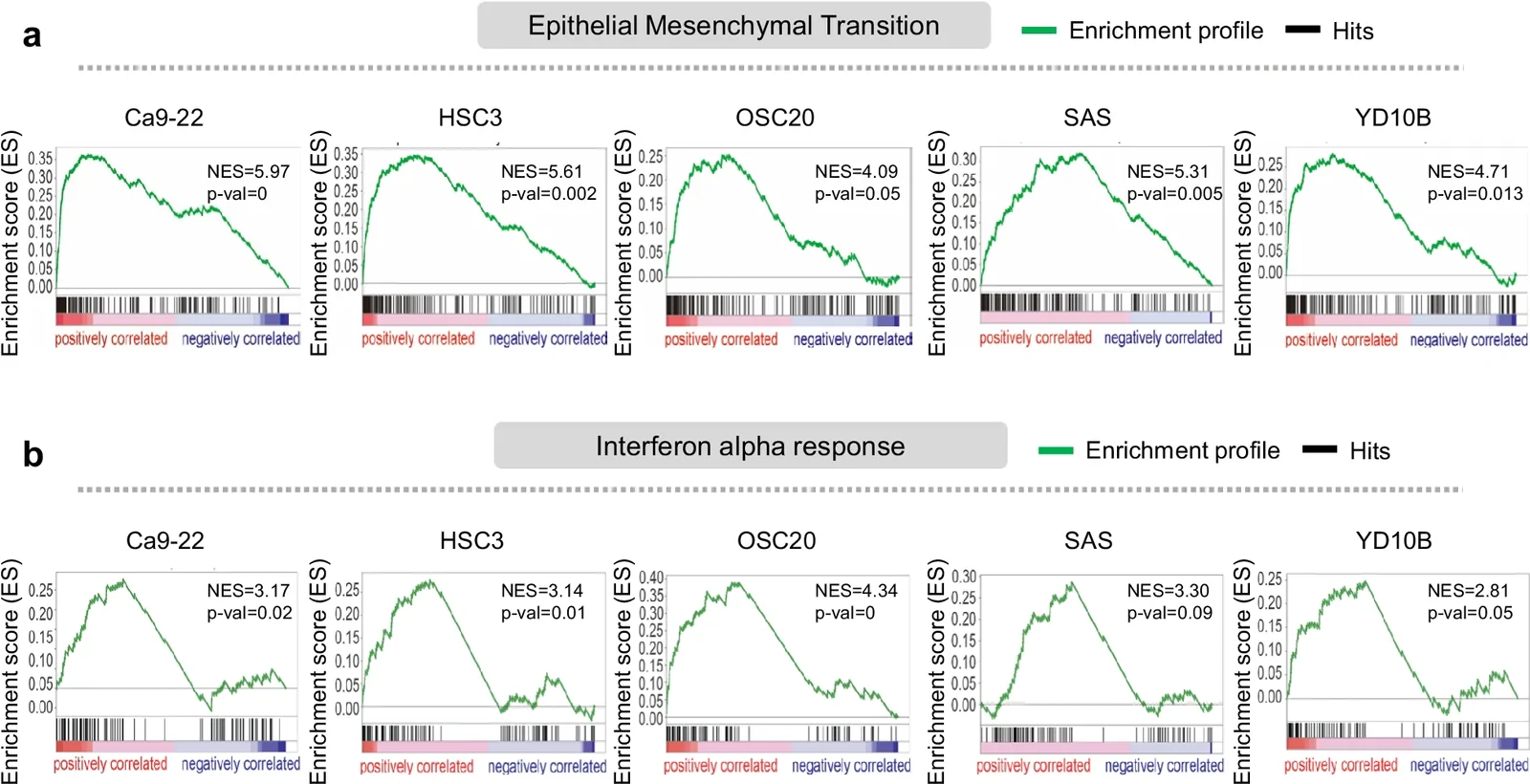
GSEA analysis of genes correlated with re-expressed by DNMT1 inhibitor (5-aza-dC). GSEA revealed the hallmarks of EMT and interferon alpha response positively correlated with gene sets by DNMT1 inhibitor (5-aza-dC). Nominal p value calculated by the permutation test in GSEA analysis. NES indicates normalized enrichment score

The GSEA findings provide important biological context for the five candidate genes identified in this study. The significant enrichment of the EMT pathway among genes reactivated by 5-aza-dC treatment is consistent with the known roles of *GPX3, ANG, CTGF, GPRC5B*, and *BAMBI* in regulating cell adhesion, extracellular matrix remodeling, and tumor invasiveness, suggesting that their coordinated epigenetic silencing may collectively facilitate EMT activation and thereby contribute to the invasive phenotype of OSCC. Notably, *CTGF* is a well-established regulator of EMT-associated processes, and its promoter hypermethylation-driven silencing in OSCC, as demonstrated in the present study, may represent a key epigenetic event that removes a critical brake on EMT progression in oral cancer (Fig. 8a). The enrichment of the interferon alpha response pathway further suggests that epigenetic silencing of these candidate genes may additionally impair anti-tumor immune surveillance in OSCC, a finding with potential implications for predicting response to immune checkpoint inhibitor therapy in this disease (Fig. 8b). While direct functional validation of these pathway-level observations remains to be performed, these GSEA data collectively suggest that the promoter hypermethylation-driven silencing of the identified candidate genes is not an isolated epigenetic event but rather a functionally coordinated mechanism contributing to multiple hallmarks of OSCC malignancy. The biological and clinical implications of these findings are further elaborated in the following discussion.

## Discussion

Aberrant promoter CpG island DNA hypermethylation is one of the most prevalent epigenetic alterations in human cancer, contributing to transcriptional silencing of tumor suppressor genes across virtually all tumor types [21]. The cancer DNA hypermethylome is often tumor type-specific, arises early in neoplastic transformation, and has proven instrumental in identifying novel methylation biomarkers and putative tumor suppressor genes with translational utility extending from diagnostics to predicting therapeutic response [22–24]. Despite this, comprehensive epigenomic characterization remains lacking for oral cancer. In the present study, we addressed this gap by applying a genome-wide pharmacologic demethylation strategy coupled with transcriptomic profiling across multiple OSCC cell lines, representing one of the first comprehensive efforts to define the promoter CpG island DNA hypermethylome in oral cancer. Through this stepwise discovery and validation pipeline, we identified five candidate genes, *GPX3, ANG, CTGF, GPRC5B*, and *BAMBI*, demonstrating consistent cancer-specific promoter hypermethylation-associated silencing in OSCC, each validated through multiple orthogonal approaches including RT-PCR, MSP, bisulfite sequencing, IHC, and large-scale TCGA-HNSC based analysis. Among the five candidate genes, *CTGF* promoter hypermethylation showed the most clinically significant association, with poor overall survival observed in our institutional cohort (p=0.030). The remaining four genes did not reach statistical significance in the institutional cohort, likely reflecting the combined influence of reduced statistical power, platform-specific differences between binary MSP and continuous Illumina HumanMethylation450 array β-values, and population-level heterogeneity between the single-center institutional cohort and the multi-institutional TCGA dataset. Importantly, multivariable Cox regression in the TCGA cohort did not confirm *CTGF* or any gene combination as an independent prognostic factor after adjustment for clinical covariates, and these survival findings should therefore be considered exploratory, warranting prospective validation in larger, well-annotated cohorts.

While *CTGF* demonstrated the most consistent individual prognostic significance, the remaining four genes collectively represent a biologically meaningful set of epigenetically silenced loci, supporting our proposal that a multi-gene methylation panel may offer superior prognostic stratification compared to any single gene alone. Consistent with this rationale, systematic evaluation of multi-gene combinations identified *ANG+CTGF* as the only combination demonstrating statistically significant and reproducible prognostic associations across both cohorts (institutional cohort p=0.047, Fig. S4e; TCGA p=0.013, Fig. 7c). Given the shared involvement of *ANG* and *CTGF* in angiogenesis and extracellular matrix remodeling, processes central to OSCC invasion, their concurrent silencing may more precisely capture the aggressive tumor phenotype than either gene alone, and prospective validation of this two-gene panel warrants prioritization in future studies. Similar multi-marker biomarker strategies have proven successful in other solid tumors; for instance, several non-invasive liquid biopsy biomarkers have been developed for prostate cancer to overcome the low specificity of PSA-based screening [25]. This precedent supports the translational potential of our multi-gene methylation panel as a non-invasive biomarker strategy for OSCC.

*CTGF*, also known as *CCN2*, is a matricellular protein involved in angiogenesis, osteogenesis, and extracellular matrix remodeling [26, 27]. Its role in cancer is notably context-dependent and has remained a long-standing source of controversy: *CTGF* promotes tumor growth and metastasis in breast cancer, glioblastoma, and esophageal adenocarcinoma [28–31], yet functioning as an anti-growth and anti-metastatic factor in breast, colon, and non-small cell lung cancers [32–34]. Our findings support a tumor-suppressive role for *CTGF* specifically in oral cancer, although the molecular basis of this tissue-specific duality warrants further investigation. *CTGF* exhibits context-dependent, dual roles across cancer types, promoting growth and metastasis in breast, brain, and esophageal cancers [26], while inhibiting metastasis in colon and lung cancers [32, 35–38] and the biological basis for this tissue-specific switch between oncogenic and tumor-suppressive activity remains unresolved. This controversy is mirrored in OSCC, where conflicting reports have shown both inhibition of tumor growth [39] and a link between *CTGF* and bony destruction [40], leaving its precise role unclear. Notably, a recent study directly addressed this ambiguity by demonstrating that *CTGF* suppresses migration and invasion in OSCC through downregulation of miR-504 via a *FOXP1*-dependent pathway [41], providing direct mechanistic evidence consistent with a tumor-suppressive function of *CTGF* in oral cancer specifically. Our findings, demonstrating promoter hypermethylation-driven silencing of *CTGF* as a robust and reproducible prognostic marker across two independent cohorts, are highly consistent with this tumor-suppressive role and further support that the oncogenic versus tumor-suppressive duality of *CTGF* is governed by tissue-specific context, the molecular basis of which warrants further investigation.

Finally, GSEA data provided important biological context for the broader OSCC hypermethylome. Genes reactivated by pharmacologic demethylation were significantly enriched for hallmark cancer pathways, most notably EMT and interferon alpha response, suggesting that promoter hypermethylation in OSCC reflects a coordinated mechanism suppressing functionally relevant tumor suppressive programs rather than a random epigenetic event. The EMT enrichment is consistent with the known role of epigenetic dysregulation in tumor invasion and metastasis, while enrichment of the interferon alpha response pathway suggests a potential role for epigenetic silencing in immune evasion, with implications for epigenetic immunotherapy. While these pathway-level findings require further functional validation, they collectively support the OSCC DNA hypermethylome as a biologically coherent set of alterations shaping tumor progression.

The present study has several limitations that should be acknowledged. A key limitation concerns the prognostic validation of our candidate genes: while *CTGF* promoter hypermethylation was significantly associated with overall survival in our institutional cohort (p=0.030), this association was not confirmed in the TCGA HNSC cohort under multivariable Cox regression adjusting for age, sex, and tumor stage, nor across sensitivity models incorporating additional clinical covariates including HPV status, smoking history, and nodal status; this null result also extended to *ANG, GPX3*, and all gene combination panels tested. We therefore do not consider these genes independently validated cross-cohort prognostic biomarkers at this stage, and prospective validation in larger cohorts using prespecified cutoffs and full covariate adjustment will be necessary to establish their clinical utility in OSCC. Additionally, while the present study establishes cancer-specific promoter hypermethylation of *GPX3, ANG, CTGF, GPRC5B*, and *BAMBI* as a consistent epigenetic feature of OSCC, the biological and functional roles of these genes in oral carcinogenesis remain to be experimentally defined. We hypothesize, based on their identification through genome-wide DNA hypermethylome profiling and their consistent association with patient outcomes, that these genes may function as novel tumor suppressors in oral cancer, and validating this hypothesis through functional studies, including gene knockdown, overexpression, and rescue experiments, represents a compelling next step building directly on the present findings. Taken together, this study provides the first comprehensive characterization of the OSCC DNA hypermethylome, laying the groundwork for both mechanistic investigation and clinical translation in the management of oral cancer, with prospective functional and clinical validation studies representing the most important future directions.

## Conclusion

Our data reveal one of the first systematic genome-wide characterizations of the promoter CpG island DNA hypermethylome in OSCC, employing a pharmacologic demethylation strategy coupled with transcriptomic profiling to identify and validate cancer-specific epigenetically silenced genes. Through a rigorous stepwise discovery and validation pipeline spanning in vitro cell line models, primary clinical specimens, and large-scale independent cohort analysis using the TCGA HNSC dataset, we identified five candidate genes, *GPX3, ANG, CTGF, GPRC5B,* and *BAMBI*, as consistently hypermethylated and transcriptionally silenced in oral cancer. Among these, *CTGF* promoter hypermethylation emerged as the most clinically significant finding, demonstrating reproducible association with poor overall survival across two independent cohorts, while pathway enrichment analysis implicated the broader OSCC hypermethylome in the coordinated epigenetic suppression of functionally relevant tumor suppressive programs including EMT and interferon alpha response pathways. Collectively, these findings advance our understanding of the epigenetic landscape of OSCC tumorigenesis and provide a foundation for the development of a clinically applicable multi-gene DNA methylation biomarker panel for OSCC detection, prognostication, and epigenetic therapeutic targeting. Future functional validation and prospective clinical evaluation will be essential to fully realize the translational potential of these epigenetic biomarker candidates for clinical implementation in oral cancer.

## Materials and methods

### Cell culture and treatment

Ten human OSCC cell lines (Ca9-22, HSC4, OSC20, SAS, SCC25, HN22, YD10B, YD38, YD9, and HSC3) were maintained in appropriate media supplemented with 10% fetal bovine serum and 1% antibiotic-antimycotic solution at 37 °C in 5% CO2, as previously described [20]. For epigenetic treatment, cells were exposed to 5 μM 5-aza-2′-deoxycytidine (5-aza-dC; Sigma-Aldrich) for 72 hours and trichostatin A (TSA; Sigma-Aldrich) for 18 hours.

### RNA extraction and quantitative RT-PCR

Total RNA was extracted from all cell lines using the RNeasy Mini Kit (QIAGEN, Germantown, USA) and reverse-transcribed into first-strand cDNA using the SuperScript II First-Strand Synthesis Kit (Invitrogen, Carlsbad, USA). Quantitative RT-PCR was performed on a CFX96™ Real-Time PCR Detection System (Bio-Rad) using SYBR Green Master Mix (Thermo Fisher Scientific) as previously described [20]. Gene expression levels were normalized to GAPDH using the 2-ΔΔCt method, and primer sequences are listed in Supplementary Table S5.

### RNA-seq and gene expression analysis

We analyzed five human oral squamous cell carcinoma (OSCC) cell lines: Ca9-22, HSC-3, OSC-20, SAS, and YD-10B. Cells were treated with 5-aza-dC, TSA, or untreated control cells (Mock), with two replicate RNA-seq libraries for each condition in each cell line. Total RNA was extracted from each sample, and RNA-seq libraries were prepared and sequenced on an Illumina platform. Low-quality reads and adapter sequences were removed using Trim Galore. Sequencing reads were aligned to the human reference genome (GRCh38/hg38) using STAR [42]. Transcripts were assembled with Cufflinks, and gene expression levels were estimated as fragments per kilobase of transcript per million mapped reads (FPKM). Read depth and alignment quality metrics are summarized in Table S6. Differential expression was analyzed separately for each cell line using Cuffdiff by comparing Mock cells with 5-aza-dC-treated cells (M vs. A) and Mock cells with TSA-treated cells (M vs. T). The results of the differential expression analysis for each cell line (log2 fold change and q-values), along with gene-level FPKM values for Mock-treated, TSA-treated, and 5-aza-dC-treated cells, were compiled into a single dataset and deposited in Zenodo (https://doi.org/10.5281/zenodo.19569589) in XLSX format. Across the 30 RNA-seq libraries, total reads ranged from 27.8 to 45.2 million per sample, and uniquely mapped reads ranged from 91.0% to 93.9%. No separate batch correction was applied because all RNA-seq libraries were processed using the same library-preparation and sequencing workflow, and no independent batch variable was available for modeling. The RNA-seq experiment was used as an exploratory screening step, and candidate genes were subsequently evaluated by qRT-PCR, MSP, bisulfite sequencing, IHC, and TCGA analyses. For downstream analysis, only protein-coding genes were included. Genes with an absolute log2 fold change of at least 0.585, corresponding to a 1.5-fold change relative to Mock, were selected. To identify genes commonly induced by demethylation treatment, genes upregulated by 5-aza-dC in each cell line were compared across the five OSCC cell lines. CpG islands and promoter regions were annotated based on the hg38 reference genome to examine the relationship between promoter CpG features and treatment-associated changes in gene expression.

### DNA Methylation analyses

Genomic DNA was extracted using a standard phenol-chloroform method and bisulfite-converted using the EZ DNA Methylation Kit (Zymo Research, Irvine, USA). Methylation-specific PCR (MSP) and bisulfite sequencing were performed as previously described [43]. For bisulfite sequencing, at least five clones per locus were randomly selected and sequenced on an ABI3730xl DNA analyzer to determine CpG methylation patterns. PCR conditions and primer sequences are provided in Supplementary Table S5.

### Tissue Samples

For OSCC samples, paraffin-embedded tissue specimens from patients who were diagnosed and surgically treated at the Department of Oral and Maxillofacial Surgery, Pusan National University Dental Hospital, between 2009 and 2014 were retrieved. For normal oral mucosal tissue for comparison, fresh healthy tissue that had been obtained during dental procedures, such as gingivoplasty, third molar extraction, or implant surgery, was used. Clinicopathologic features of total oral cancer patient samples including age, gender, histopathologic grade, and the TNM stage, was previously reported [20]. In contrast to the earlier study, the present work utilizes this cohort to validate novel candidate genes identified through transcriptome-based screening. Thus, the current analysis addresses a distinct research question and employs independent experimental and analytical approaches. This study was approved by the Institutional Review Boards (IRB) of Pusan National University Dental Hospital (IRB No. PNUDH-2019-046) and written informed consent was obtained from all patients.

### TCGA-HNSC derived oral cancer patient cohort for mRNA expression and DNA methylation validation

TCGA validation and survival analysis processed, de-identified TCGA-HNSC molecular and clinical data were used for TCGA validation. The dataset corresponded to the TCGA Head and Neck Squamous Cell Carcinoma project (TCGA-HNSC) in cBioPortal (https://portal.gdc.cancer.gov/) [44], Firehose Legacy (`hnsc_tcga`), and the local curated workbook was generated from the public source on June 19, 2019. Controlled-access TCGA files were not used. The matched expression and methylation frame contained 528 primary HNSC tumors. After anatomic curation, 344 oral-cavity cases were retained for the OSCC-focused analysis (Table S4); 184 non-oral or non-assignable cases were excluded, including larynx (n = 116), tonsil (n = 45), hypopharynx (n = 10), oropharynx (n = 9), lip (n = 3), and site-not-assignable cases (n = 1). For gene-level methylation-expression analyses, *CTGF* and *GPX3* methylation used cBioPortal gene-level HM450 values, whereas ANG methylation was represented by the mean beta value across all ANG-associated HM450 probes because ANG was not available in the cBioPortal gene-level HM450 output. Matched methylation and expression values were available for 339 retained cases. Overall survival was measured in months from TCGA clinical time-to-event fields. One retained oral-cavity case lacked overall-survival time, leaving 343 cases for Kaplan-Meier visualization; reverse Kaplan-Meier median follow-up was 34.3 months. For Fig 7, the representative best CpG for each gene was fixed before survival analysis as the HM450 probe with the lowest tumor-versus-normal methylation Mann-Whitney P value within the TSS-centered promoter window: *CTGF* cg24684168 (+/−1000 bp), *ANG* cg10850001 (+/−100 bp), and *GPX3* cg26638444 (+/−1000 bp). Gene-combination scores were calculated as the arithmetic mean of the selected beta values, and high and low groups were defined by the cohort median.

### Immunohistochemistry (IHC)

Immunohistochemical staining was performed on primary OSCC tumor tissues (n=4) and matched normal oral mucosal tissues (n=4) from our institutional cohort, as previously described [20]. Primary antibodies against GPX3 (1:100; AF4199; R&D Systems), ANG (1:200; AF265; R&D Systems), CTGF (1:100; 23936-1-AP; Proteintech), GPRC5B (1:500; NBP1-87160; Novus), and BAMBI (1:100; AF921; R&D Systems) were applied using a Benchmark autostainer with an I-View detection kit (Ventana Medical Systems, Tucson, USA). Sections were counterstained with hematoxylin, dehydrated, and cleared in xylene.

### Gene set enrichment analysis (GSEA)

GSEA was performed using the Broad Institute GSEA software with gene sets from the Molecular Signatures Database (MSigDB). All protein-coding genes from the Cuffdiff output were ranked using the following metric: log2(fold change) × −log10(q-value +1.0 × 10⁻^12^). This ranking scheme was used to reflect both the direction and magnitude of expression change as well as its statistical significance. The resulting pre-ranked gene list was used as input for GSEA. The normalized enrichment score (NES) was used to compare enrichment strength after accounting for differences in gene-set size and enrichment-score scaling.

### Statistical analysis

All experimental data are reported as mean ± standard deviation, and all experiments were repeated three times. P‐values which were less than 0.05 were considered significant. GraphPad Prism software 9.0 was used for statistical analysis. For survival analysis, each methylation level was entered as a continuous covariate in a univariable Cox proportional-hazards model (Efron's approximation for tied event times; lifelines 0.30.3).

## Supplementary Information

Supplementary Material 1.

Supplementary Material 2.

## Data Availability

The datasets generated and analyzed in the study were compiled into a single dataset and deposited in Zenodo (https://doi.org/10.5281/zenodo.19569589) in XLSX format. All data generated and analyzed during this research are included in this article and available upon request.

## Abbreviations

5-aza-dC: 5-aza-2′-deoxycytidine
ANG: Angiogenin
BAMBI: BMP and activin membrane-bound inhibitor
CTGF: connective tissue growth factor
DKO: Double knockout
DNMT: DNA Methyltransferase
EMT: Epithelial-Mesenchymal Transition
FDR: False discovery rate
FPKM: Fragments per kilobase of transcript per million mapped reads
GPX3: Glutathione peroxidase 3
GPRC5B: G protein-coupled receptor family C group 5 member B
GSEA: Gene set enrichment analysis
HDAC: Histone Deacetylase
HNSC: Head and neck squamous cell carcinoma
IHC: Immunohistochemistry
KM: Kaplan-Meier
MSigDB: Molecular Signatures Database
MSP: Methylation specific polymerase chain reaction
NES: Normalized enrichment score
OSCC: Oral Squamous Cell Carcinoma
qRT-PCR: Quantitative real-time reverse transcription polymerase chain reaction
RNA-seq: RNA sequencing
SD: Standard deviation
TCGA: The Cancer Genome Atlas
TSA: Trichostatin A
TSG: Tumor suppressor gene
TSS: Transcription start site
