# Supplementary material for: Transcriptome-based epigenetic screening identifies DNA hypermethylation signatures as prognostic biomarkers in oral squamous cell carcinoma
Source: Mol Biomed. 2026 Sep 17;7:173. doi: 10.1186/s43556-026-00584-4 (PMC13582760; doi:10.1186/s43556-026-00584-4)
Supplement: Supplementary file 1 [file 43556_2026_584_MOESM1_ESM.docx]

Supplementary Materials for

**Transcriptome-Based Epigenetic Screening identifies DNA Hypermethylation Signatures as Prognostic Biomarkers in Oral Squamous Cell Carcinoma**

Yu Kyeong Han ^1,2,†^, Jihye Park ^3,†^, Hye Su Han ^1,2^, Keunsoo Kang ^3^, Hae Ryoun Park ^4^ , Joo Mi Yi ^1,2,*^

*Correspondence: jmyi76@inje.ac.kr

**This file includes:**

- Supplementary Materials and Methods
- Figure S1-S7

Supplementary Materials and Methods

*Primer design*

Primers for reverse transcription PCR (RT-PCR) expression analysis were designed using the open-access Primer3 program (http://bioinfo.ut.ee/primer3/). Primers for methylation-specific PCR (MSP) analysis of the candidate gene promoter regions were designed using MSPprimer (<http://www.mspprimer.org>). All primer sequences are provided in Table S5.

*TCGA-HNSC derived oral cancer patient cohort for mRNA expression and DNA methylation validation*

For Cox analyses, methylation scores were modeled continuously per 0.1 beta-value increase. The primary adjusted model included age at diagnosis per decade, sex, and pathologic stage III/IV versus I/II. Sensitivity models additionally considered HPV status, smoking history, nodal status, T category, combined HPV/smoking/nodal adjustment, and primary radiation. A stricter anatomic-site exclusion check was performed separately to assess residual site heterogeneity. Treatment variables were incomplete and heterogeneous in timing; therefore, treatment was not included in the primary model, and primary radiation was examined only as a sensitivity model. Proportional-hazards assumptions were checked using rank-based Schoenfeld residual tests.

*Statistical analysis*

Proportional-hazards behavior of the reported configurations was confirmed by the scaled Schoenfeld-residual test. Kaplan-Meier curves were drawn after median-split of the MRS and the difference between strata was assessed by a two-sided log-rank test. Discrimination was quantified by Harrell's concordance index in 5-fold cross-validation repeated over 10 random seeds, with Cox ridge penalty 0.01 for numerical stability. The enumeration-level multiple-testing burden was controlled by the Benjamini-Hochberg step-up procedure applied to the 31 Cox P-values.

**Fig. S1.** Schematic representation of CpG islands at the promoter regions of *GPX3, ANG, CTGF, GPRC5B,* and *BAMBI*. Blue and green bars indicate primer locations for methylation-specific PCR (MSP) and bisulfite sequencing (BSQ), respectively. Orange vertical lines indicate individual CpG sites. TSS, transcriptional start site. These data correspond to Fig. 4.

**Fig. S2.** Correlation between promoter DNA methylation and gene expression levels of candidate genes in the TCGA HNSCC dataset. Negative correlation between gene expression and promoter methylation of (a) GPX3 (Spearman ρ = −0.621, p = 1.53 × 10⁻³⁷), (b) CTGF (Spearman ρ = −0.155, p = 4.32 × 10⁻³), and (c) GPRC5B (Spearman ρ = −0.384, p = 2.32 × 10⁻¹³) was observed using the data from TCGA HNSCC database. (d) BAMBI and (e) ANG did not reach statistical significance in the correlation analysis. These data correspond to Fig. 5.

**Fig. S3.** Protein expression of five genes in primary OSCC tumors and normal oral mucosa tissue samples. Immunohistochemical analysis of additional samples (n=2) showing the expression of GPX3, ANG, CTGF, GPRC5B, and BAMBI in primary OSCC tissues and normal oral mucosa. Left images: × 20, scale bar, 200 µm; Right images: × 40, scale bar, 100 µm. These data correspond to Fig. 6.

**Fig. S4.** Kaplan-Meier curves showing the effect of candidate gene methylation on overall survival in institutional cohort and TCGA HNSCC dataset. (a-c) DNA methylation and overall survival among patients with OSCC (n=343) from the HNSCC TCGA dataset. Survival curves comparing high (red, n=172) versus low (blue, n=171) expression groups for (a) GPX3, (b) BAMBI, and (c) GPRC5B. None of these genes showed statistically significant associations with overall survival (GPX3: log-rank p=0.7811; BAMBI: log-rank p=0.3862; GPRC5B: log-rank p=0.9022). (d-i) DNA methylation and overall survival among patients with OSCC from the institutional cohort (n=102). Survival curves comparing unmethylated (blue) versus methylated (magenta) groups. Single gene analysis showed significant association for (d) CTGF methylation with overall survival (p=0.030). Two-gene methylation combinations showed significant associations: (e) ANG+CTGF (p=0.025), (f) GPRC5B+CTGF (p=0.044), and (g) ANG+GPRC5B (p=0.048). (h) Three-gene combination ANG+GPRC5B+CTGF (p=0.054) and (i) four-gene combination ANG+GPRC5B+CTGF+BAMBI (p=0.058) did not reach statistical significance. These data correspond to Fig. 7.

**Fig. S5. Gene-level methylation-expression and promoter-window context for CTGF, ANG, and GPX3 in the curated oral-cavity TCGA-HNSC cohort.** (A) Gene-level HM450 methylation versus RNA expression for CTGF, ANG, and GPX3. CTGF and GPX3 methylation used cBioPortal gene-level HM450 values. Because ANG was not available in the cBioPortal HNSC gene-level HM450 output, ANG methylation was calculated as the mean beta value across all ANG-associated HM450 probes. Spearman correlations were CTGF rho = -0.402 (95% CI -0.488 to -0.309, P = 1.36 x 10^-14; n = 339), ANG rho = 0.060 (95% CI -0.047 to 0.165, P = 0.2704; n = 339), and GPX3 rho = -0.621 (95% CI -0.682 to -0.551, P = 1.53 x 10^-37; n = 339). (B) Nominal Cox P values across TSS-centered promoter windows for average promoter beta values and BEST CpG features. (C) Corresponding within-screen Cox FDR q values. Dashed and dotted reference lines indicate 0.05 and 0.10, respectively. BEST CpG features were fixed before survival analysis as the CpGs with the lowest tumor-versus-normal methylation Mann-Whitney P values within each promoter window, not by survival P values.

**Fig. S6. Stage-, age-, and sex-adjusted continuous methylation Cox models and clinical covariate availability in the curated oral-cavity TCGA-HNSC cohort.** (A) Primary adjusted Cox proportional hazards models for continuous CTGF, ANG, GPX3, ANG+CTGF, GPX3+CTGF, and GPX3+ANG+CTGF methylation scores. Hazard ratios are shown per 0.1 increase in methylation score and were adjusted for pathologic stage III/IV versus I/II, age per decade, and sex. Estimates were CTGF HR = 1.06 (95% CI 0.92-1.22, P = 0.4132), ANG HR = 1.03 (95% CI 0.53-1.99, P = 0.9397), GPX3 HR = 1.02 (95% CI 0.94-1.12, P = 0.5975), ANG+CTGF HR = 1.11 (95% CI 0.85-1.44, P = 0.4323), GPX3+CTGF HR = 1.07 (95% CI 0.92-1.25, P = 0.3567), and GPX3+ANG+CTGF HR = 1.11 (95% CI 0.89-1.39, P = 0.3610; all n = 311, events = 143). (B) Sensitivity Cox models for continuous methylation scores in the curated oral-cavity TCGA-HNSC cohort. Forest plots show univariable and covariate-adjusted Cox proportional hazards models for CTGF, ANG, GPX3, ANG+CTGF, GPX3+CTGF, and GPX3+ANG+CTGF methylation scores. Hazard ratios are shown per 0.1 increase in methylation score, and row labels provide the model-specific sample size and event count. The primary adjusted model included pathologic stage III/IV versus I/II, age per decade, and sex and corresponds to Figure SYA. Additional exploratory models considered stage only, stage plus age, stage plus sex, age plus sex, HPV status, smoking history, nodal status, T category, combined HPV/smoking/nodal adjustment, and primary radiotherapy. These sensitivity analyses did not identify a consistent independent methylation-survival association.

**Fig. S7. Availability of clinical covariates in the retained oral-cavity cohort.** Orange bars indicate covariates included in the primary adjusted Cox model; blue-gray bars indicate additional clinical variables considered in sensitivity analyses. All Cox analyses are exploratory and use continuous methylation scores to avoid data-driven survival cutoff selection.
